# Living Life to the Fullest After Organ Transplantation

**DOI:** 10.3389/ti.2026.16595

**Published:** 2026-05-12

**Authors:** Andrea Zajacova, Coby Annema, Nina Pilat, Thierry Berney

**Affiliations:** 1 Editorial Fellow, Transplant International; 2 BREATHE, KU Leuven, Leuven, Belgium; 3 Department of Health Sciences, Section of Nursing Science, University Medical Center Groningen, Groningen, Netherlands; 4 Deputy Editor-in-Chief, Transplant International; 5 Department of Cardiac and Thoracic Aortic Surgery, Medical University of Vienna, Vienna, Austria; 6 Editor-in-Chief, Transplant International; 7 Division of Transplantation, Immunology and Nephrology, Hospices Civils de Lyon, Lyon, France

Ensuring a good quality of life after organ transplantation is a priority that all transplant physicians, surgeons and healthcare professionals should strive to achieve for their patients. Indeed, organ transplantation is not simply about surviving and the ability to participate in meaningful activities of life is of critical importance [1]. This should be a self-evident fact, but the journey to overcome complications, co-morbidities, medication side-effects, and treatment burden, in order to achieve the desired physical, mental, and social outcomes, can be a bumpy ride, and has been the topic of a recent special issue of *Transplant International* titled “Living well after transplantation” [1, 2].

Living well after transplantation (arguably, the ability to participate in normal life activities) is by definition a subjective concept and assessing it can only be done from the patient perspective, and has been understudied. The development and implementation of patient-reported outcome measures into clinical practice and clinical trials is the first step ensuring the patient voice is heard systematically [3].

To have a meaningful life after a solid organ transplant, patients can use their improved health status to once again enjoy time with family and friends, to travel and to return to work [4]. To achieve this, healthcare providers should look beyond medical support in enhancing long-term wellbeing, and most importantly, organ recipients should see themselves as creators of their own wellbeing [5].

The ability to practice sport and physical activity is a significant part not only of a return to social life but also of health-related quality of life [6]. Indeed, the effect of the quantity of sport activity was significant on the General Health and Role Emotional components of the SF-36 questionnaire, with more sport activity associated with higher HRQoL [7].

Sport and physical activity are increasingly recognized as important components of recovery and long-term quality of life after lung transplantation. Evidence from reviews and interventional studies suggests that exercise training before and after transplantation can improve or preserve exercise capacity, muscle strength, functional status and health-related quality of life in lung transplant recipients [8, 9]. Structured rehabilitation programs and targeted interventions, including high-intensity interval training, have been shown to be feasible and safe while contributing to improvements in aerobic capacity and muscular strength [10, 11]. In addition, studies examining symptom responses during exercise have provided insight into ongoing limitations such as dyspnea, effort perception, and muscle discomfort that may be experienced by lung transplant recipients [12]. Importantly, evidence from cohorts of transplanted athletes indicates that some are able to participate in recreational or competitive sport, demonstrating substantial physical recovery and highlighting the potential for lung transplant recipients to regain high levels of physical functioning and social participation [13, 14].

The letter published in this issue of *Transplant International* is an impressive report of how far organ transplant recipients are able to challenge themselves from the physical standpoint and even achieve feats that most individuals from the general population would be unable to achieve [15]. The transplantation team from the Medical University of Vienna enrolled 9 lung transplant recipients in a high-altitude mountaineering expedition, in which one patient was able to summit Mount Aconcagua, the highest peak in the Andes, without supplemental oxygen. The letter also demonstrates the responsibility and the cautiousness of the medical team, who took great care to acclimatize and monitor their patients, and thus avoid the potential detrimental outcomes of extreme physical activity after organ transplantation [6].

Beside this exceptional achievement from the medical, physical and emotional standpoints, there is a clear symbolic value in the image of a lung transplant recipient climbing to extreme altitudes without supplemental oxygen support. If anything, this experience is an staggering and thrilling demonstration that organ transplant recipients can not only “live well” but also live their lives to the fullest.
